# Using Bourdieu to explore graduate attributes in two online Master’s programmes

**DOI:** 10.1007/s10459-019-09885-6

**Published:** 2019-03-26

**Authors:** Gillian Aitken, Derek Jones, Tim Fawns, Douglas Sutherland, Sarah Henderson

**Affiliations:** Edinburgh Medical School, Chancellor’s Building, Little France Crescent, Edinburgh, EH16 4SB UK

**Keywords:** Online, Postgraduate, Bourdieu, Graduate attributes

## Abstract

Within the expansion of postgraduate educational qualifications for health professionals, graduate attributes have become important markers of outcomes and value. However, it is not clear how or when graduate attributes develop, or how they are applied in professional practice after graduation. We interviewed 17 graduates from two online Master’s programmes to explore their perceptions of how postgraduate study had influenced their practice and professional identity. Our thematic analysis produced three main themes (academic voice, infectious curiosity, and expanding worldview) which reflected changes in the participants’ confidence, attitude, perspective, and agency across professional and academic settings. We then conducted a secondary phase of analysis using Bourdieu’s concepts of ‘field’, ‘capital’, and ‘habitus’. While graduate attributes have been conceptualised as the context-independent acquisition of traits that can be employed by individuals, Bourdieu’s framework highlights their relational qualities: they are caught up in the cultural history and context of the student/professional, the reputation of the awarding institution, and the graduate’s location within a network of professional peers.

## Introduction

Taught postgraduate (PGT) degrees are increasingly seen by clinicians as a way of gaining additional skills key to future career development. This is reflected in a proportionately greater increase in PGT enrolments related to healthcare and education (Universities UK 2018). Many programmes are offered online, affording busy clinicians the opportunity to study part-time while continuing to work, and providing the potential for engagement with an international, multi-disciplinary academic community. In the increasingly crowded and expansionist higher education sector, academic institutions attempt to articulate what is unique about their offerings. Graduate attributes—those allegedly transferable skills and dispositions produced through engagement in programmes of study (Hager, 2006)—are referred to in the strategic plans of most universities. However, they are a contentious topic in the literature, with debate focusing on whether these attributes really can be transferred from one setting to another (e.g. from University to workplace), and whether the development of attributes should be considered a product that can be measured, or a developmental process (Holmes 2013).

Online PGT programmes are well suited to exploring the transferability of academic learning to the clinical workplace. Many students study part-time while working, creating opportunities to test their learning in practice, and then returning to share such experiences with the academic community. It is surprising, then, that little has been published on the experiences and motivations of clinicians engaged in PGT programmes. Obtaining a Master’s degree is seen as desirable in healthcare education leaders (Tekian and Harris 2012) and such qualifications are often cited as essential in job specifications suggesting that graduates of such programmes go on to undertake influential roles (Steinert 2012; Sethi et al. 2018). Sethi et al. (2016) and Cunningham and Dovey (2016) surveyed graduates from medical education and general practice programmes respectively, indicating changes in practice, self-efficacy, critical thinking and sense of belonging to an academic community. Sethi et al. (2018) reported that, independently of any gains in knowledge, the medical education postgraduate qualification itself was seen to aid career progression, indicating a potentially complex relationship between the benefits of what was learned on the programme and the status of the award.

### Graduate attributes at the University of Edinburgh

The work of Sethi et al. (2018), mentioned above, leads us to further consider the different ways in which PGT programmes can create value and change in practice, identity and agency for graduates. Hager (2006) suggested that graduate attributes should not be viewed as homogenous, discrete entities that are acquired fully-formed, but as integrated elements of identity or practice that are modified and refined over time. As such, we were interested to explore how such attributes might evolve during and after programmes of postgraduate study, and how they manifest in professional practice. Like many higher education institutions, the University of Edinburgh lists a Graduate Attributes Framework containing a set of mindsets and skills that make up “What [it means] to be a University of Edinburgh graduate,” and that “set them apart” from students and graduates of other institutions (UoE 2016). They are applicable across all undergraduate and postgraduate programmes in all disciplines. We have listed them below as they appear on the University website.

University of Edinburgh graduates have:

- curiosity for learning that makes a positive difference
- courage to expand and fulfil their potential
- passion to engage locally and globally

University of Edinburgh graduates are:

- creative problem solvers and researchers
- critical and reflective thinkers
- effective and influential contributors
- skilled communicators

These attributes are categorised into things that graduates *have* and things that they *are*. In other words, these graduate attributes are portrayed as possessions or states of being; they are acquisitional or transformative. It is not clear to us how or when they are realised, or how this relates to curricular design. To better understand how these graduate attributes manifest in practice, and how they are acquired or developed, we sought to investigate the experiences of graduates of two online PGT programmes (M.Sc. Clinical Education and MSc Management of Pain) at the University of Edinburgh. A further aim was to examine the potential of graduate attributes to transcend disciplinary boundaries and ‘transfer’ to other social, intellectual and professional contexts. To aid in this examination, we drew on the work of French sociologist Bourdieu to assist our understanding of recent graduates’ engagement in, and outcomes of, postgraduate education. Bourdieu’s (1977, 1986) concepts of field, capital and habitus allowed us to conceptualise the ways graduates felt they had developed as a result of their studies, and the perceived impact on their careers. Varpio and Albert (2013) discussed how Bourdieu’s work could be applied to medical education but, to date, a limited number of studies have done so (Hu et al. 2015; Cleland et al. 2016; Wright 2015; Brosnan 2010; Brosnan et al. 2016).

## Methods

### Sampling and recruitment

Initially, we interviewed 13 graduates from the MSc Clinical Education. We recruited those who we believed would be *information*-*rich* (Sandelowski 2000) in relation to our area of enquiry; those we thought would have interesting things to say about ways in which their learning on the programme had transformed their identity or practice. This was based on interactions of members of the research team who are also teachers on the programme (GA, DJ, TF). Within this selection, we recruited from a mixture of professions, cultural backgrounds and geographical settings, as well as what we perceived to be a range of attitudes and perspectives. Through these characteristics, and the quality of information provided, the resulting sample size provided adequate ‘information power’ (Malterud et al. 2016) to meet the aims of our exploratory research. However, as we became confident in the themes identified and the appropriateness of applying Bourdieu’s concepts, we sought further data from another programme to test our analysis. We recruited a small sample (four) of additional participants from a second programme: the M.Sc. Clinical Management of Pain. While we were not aiming to generalise about the outcomes and effects of online PGT programmes on professional practice, we thought that it would be valuable, in reflecting on the University of Edinburgh’s stated graduate attributes, to consider similarities and differences brought about by a related but different programme context and group of students. Some details of these programmes and participants are given in Table 1. The local Medical School ethics committee granted ethical approval.

**Table 1:** Participant characteristics

Participant	Programme	Discipline	Gender	Locality
1	CE	Obstetrics	F	Asia
2	CE	Nursing	F	North America
3	CE	Nurse educator	M	UK
4	CE	GP	F	Europe
5	CE	Emergency medicine	F	UK
6	CE	Clinical academic	M	North America
7	CE	Surgeon	F	Africa
8	CE	Neurologist	M	Australasia
9	CE	Nursing	M	Asia
10	CE	Lab technician	F	North America
11	CE	Emergency Medicine	M	Asia
12	CE	General medicine	F	Africa
13	CE	Nurse educator	F	UK
14	MP	Physiotherapist	M	UK
15	MP	Dentist	M	North America
16	MP	Nurse educator	F	Africa
17	MP	Anaesthetist	M	Australasia

*CE* clinical education*, MP* clinical management of pain

### Data collection

Semi-structured interviews were conducted by DS, a post-doctoral research assistant with a social science background, using online conferencing software (Blackboard Collaborate™). DS had never met any of the participants, nor had any role in either programme. Three pilot interviews (not included in the analysis) were carried out to help refine the interview questions. The interview schedule was structured around what was learned and its value in a clinical and career context. An emphasis was placed on graduate attributes, transferable skills, and career impact. Audio recordings were transcribed by a professional transcription service. Interview length ranged between 20 and 48 min, with an average of 30 min.

### Context

Both programmes studied here are delivered entirely online, using a mixture of synchronous and asynchronous communication, based on a flipped classroom approach (McLaughlin et al. 2014). Delivery of content such as recorded lectures, via the University’s virtual learning environment (VLE), is followed by discussion at videoconference tutorials and on discussion boards. Both programmes recruit participants internationally from a wide range of health professions. Both have been established for over 10 years, with over 500 graduates between them. Current student numbers are 180 for the M.Sc. Clinical Education and 75 for the M.Sc. Clinical Management of Pain.

### Data analysis

The data were thematically analysed using the process described by Braun and Clarke (2008). Initial coding from the first three interviews was carried out individually by GA, SH, and DS. There was good agreement and the resulting codes were combined and used to identify emerging themes and generate an initial coding framework. These were then compared collectively (by GA, SH, DS, and DJ), and iteratively refined across the remaining data. While the other researchers were intimately involved in the running of these programmes, DS provided a degree of externality to both data collection and analysis. There was a high degree of agreement within the coding, with minor differences resolved by discussion. The emergent themes were strongly articulated by many participants. In the second phase of analysis, these themes were viewed through the theoretical lens of Bourdieu’s concepts of field, capital and habitus (discussed in the next section), with a focus on changes in capital or habitus in relation to clinical and academic fields. The resulting framework was shared with four participants, all of whom agreed it was a good representation of their own experiences.

### Theoretical framework: Bourdieu’s field, capital and habitus

In the second phase of analysis, we used Bourdieu’s work as a theoretical framework for understanding our participants’ engagement in, and outcomes of, postgraduate education. Bourdieu used the term *habitus* to describe an individual’s unique characteristics; their tastes, perceptions, or ways of responding and thinking (Bourdieu and Wacquant 2013). Bourdieu (1977) was interested in the ways in which habitus both shapes and is shaped by practice. *Field* describes an area of practice (such as a medical school or clinical department) characterised by an internal struggle for limited power or resources (Bourdieu 1977, 1990; Bourdieu and Wacquant 2013). The distribution of power can be understood by considering the notion of *capital* or what is valued within the field (Bourdieu 1986). Capital is an acquired form of power or influence, taking many forms, all of which are ultimately resources that can be exploited. The academic skills developed during postgraduate study, and the confidence in using them, represent forms of embodied cultural capital. Other relevant examples of capital include social networks (social capital); valuable objects and materials (objectified capital), such as the qualification itself; and the association of value through the reputation of an awarding institution (institutionalised capital), such as a University.

## Results

### Participants

Seventeen graduates consented to be interviewed. To maintain anonymity, we have provided minimal biographical information (see Table 1). All participants were mid-career professionals, having been professionally qualified for at least 5 years.

### Interview themes

Our initial thematic analysis resulted in three overarching themes: academic voice, infectious curiosity and expanding worldview (summarised in Table 2). The second phase of our analysis, using the lens of Bourdieu’s concepts, showed how these themes reflected changes in habitus, underpinned by particular combinations of different but interconnected forms of capital, and how they could influence a participant’s position within their field.

**Table 2:** Themes

Academic voice	Constructing arguments
	Using a new vocabulary
	Acting with authority
Infectious curiosity	Epistemological flexibility
	Critical approach
	Knowledge consumer and producer
	Reflection
Expanding worldview	International
	Multidisciplinary
	Increased complexity

### Academic voice

An important outcome of both programmes was the development of an *academic voice*, seen as integral to the development of professional identity. This involved the acquisition of new language and an ability to synthesise material and construct coherent arguments supported by evidence. Participant 10 conveyed the distinction this could bring to professional practice.

I gained a lot of self-confidence … you know, I’m the voice of clinical education in our organisation and to say I understand the theory behind this, I know best practice… and, you know, speak with that educated voice now on that topic. (Participant 10-CE)

“Speaking the language” built confidence in discussing theory in practice contexts, and enhanced legitimacy in both academic and practice settings. Awareness and application of theory could be profoundly practical, helping participants enact change in the workplace. In combination with “up-to-date” factual knowledge, theoretical knowledge could help to “bring people on board … and inform others of what you’re trying to achieve.” (Participant 17-MP). The capacity to articulate ideas and support them with sound, evidence-based arguments went beyond language. For Participant 6, “a vocabulary, some words and concepts to describe things or think about things” enabled him to develop a “framework for thinking”. Thus, the academic voice was underpinned by new ways of seeing and thinking, and these could be very empowering. For example, Participant 15 described a greater potential for analysis and problem solving, as not only could he express himself more effectively, he could also more effectively understand others.

… There’s times that I see patients that ten years ago I would have just scratched my head at. And part of that is the didactic input that I got from going through the course. But part of it also is a new way of looking at what the patients are saying and analysing it. (Participant 15-MP).

This allowed Participant 15 to be more influential across educational and clinical fields, with colleagues, students and patients.

It allowed me to organise what I already knew in a way that I could explain it other people, and by explaining it to my classmates and to the instructors I figured out a way to explain it to the patients. (Participant 15-MP)

For Participant 4, the development of an authoritative voice supported her capacity for leadership in the practice setting.

Before the course … I would never really have offered my own opinions…in terms of how things were organised…After having done the Masters, I definitely know the most within the practice now in terms of the language of education and the theory behind it. That, for me, has given me huge confidence and definitely I’m more willing to lead and I will put myself forward and put my own ideas forward. (Participant 4-CE)

The combination of confidence, the capacity to argue persuasively, and new tools for problem solving, analysing and learning combined to support a sense of developing agency and autonomy, even in the absence of formal authority.

I’m doing a lot of new roles without necessarily the authority to impose new direction. So some of that needs to come just from convincing people that this is a good idea.” (Participant 10-CE).

### Infectious curiosity

Another important outcome that came through clearly in the data was a change in attitudes and approaches to understanding. The increased confidence described in the previous theme could produce a capacity to deal with uncertainty and complexity. This, in turn, supported participants to pursue their curiosity around previously inaccessible areas of knowledge, and to more easily question different points of view.

I suppose, by the end of the second year of the course, I was getting a bit more confident in thinking, well I don’t agree with what they said. And so I’m going to ask this question about it. (Participant 5-CE).

Participant 5 gained confidence in questioning and clarification, which was “definitely useful in medicine [where we] have to get clarification, or have a discussion about what is the right thing to do for the patient.” For Participant 14, the authority that came with the development of an academic voice was tempered by a recognised need to be open to questioning and to learning from mistakes. For her, postgraduate study had increased her awareness of the contentious nature and the personal and collective limitations of knowledge. She felt that evidence, the opinions of others, and also her own ideas, should all be subject to “external scrutiny” (e.g. by programme tutors and peers). Similarly, for Participant 13, the programme provided access to valuable, external points of view from a “learning community which was completely [outside of] the people that I would normally speak to about education stuff.”

Exposure to different ways of thinking, alternative epistemologies, different cultures and the varied practices of student peers, could provide ways into unfamiliar conceptual terrain. For Participant 4, the Clinical Education programme gave her the tools to “find her way into new areas” where “the answers aren’t so clear.” Participant 6 had deliberately chosen to pursue new ideas and perspectives through a supervised dissertation using qualitative research. Like many participants, Participant 6 had been trained in positivist scientific enquiry, but had learned to be able to see through a different epistemological perspective.

I suspect [my epistemological position has] changed, because I’m aware of alternatives. I think that alone is sufficient for it to have changed. I recognise that I do not know enough to have a firm position… My approach to the world [is now] a little bit more accepting of the idea of discourse and discursive ideas, and is much less positivistic. (Participant 6-CE)

The result was not simply that he had learned about new things but that he had developed an awareness of the uncertainty and complexity of the areas of enquiry with which he was dealing. His expanding theoretical understanding and epistemological versatility had changed his practice across multiple fields.

Having a framework to think about thinking, has helped me… manage my clinical work, possibly even my personal life, and certainly how I teach others, and how I mentor others. (Participant 6-CE)

This enhanced capacity for reflecting on practices and ideas allowed participants to rethink fundamental concepts, opening up problematic assumptions that could be examined and redressed, as discussed by Participant 15.

…the whole concept of pain. I was reaching back to: “what is pain?”… I’d never really taken the time, I guess, to examine the first assumption, and the biggest problem we as providers, and scientists in general, [have] is a failure to question our first assumptions… [Before the programme], I pretty quickly decided, “oh, I know what’s wrong there,” and that doesn’t always work out… it’s broadened my perspectives. (Participant 15-MP)

In many cases, enhanced curiosity and reflection across academic and clinical settings continued beyond graduation. This was evident in an ongoing commitment to scholarship. Beyond staying abreast of research and developments in their respective fields, many participants became involved in the active generation of new knowledge. Participant 13’s aspiration, for example, “to contribute, rather than just recite” showed a desire for agency in the construction of knowledge and advancement of her field.

We have characterised this theme as “infectious” because the desire for scholarship and knowledge spread to the participants via engagement with peers, tutors and literature, and then also from the participants to their peers and students. For example, Participant 17 spoke of new possibilities for appropriately guiding students in their own pursuit of knowledge.

The Masters has made me realise I need to step up… I’m quite keen to try and spark an interest in the trainees that I work with regarding the importance of having an inquisitive mind, and just not accepting that people do things just because, and making sure that you have an understanding why people are doing things. (Participant 17-MP)

### Expanded worldview

The theme of expanded worldview captures a change in perspective, as participants came to see the fields of healthcare and education as more complex and nuanced, with less clear-cut answers and borders. It reflects a shift from a narrow focus where specialisms, disciplines, teams, workplaces, cultures or epistemologies were often taken for granted and viewed in isolation; to a wider view in which they were seen as interacting. Both programmes recruit a range of health professionals from different disciplines and countries, resulting in wide multidisciplinary and multicultural representation in discussions. Participant 8 gave a sense of what this was like for her.

Your thinking is broadened because you are not in a four walled classroom with people who you know from your town or city…We had different accents, everybody had a different background and different experiences, so when they used to share their experiences it was very engaging … (Participant 8-CE).

Such discussion involved formulating specialised, disciplinary language into terms that could be understood by outsiders. The acquisition of new academic language and vocabulary, while allowing participants to articulate complex and nuanced concepts, also needed to be balanced by a recognition that simplified language was sometimes necessary within multidisciplinary dialogue to break down barriers to communication.

We talk about this a lot at work, saying we need the MDT approach. But, in truth, physios speak to physios and nurses speak to nurses. But, actually, what this course meant was that we had to stop using jargon. We had to actually use a language that was a little bit more accessible. (Participant 14-MP)

At the same time, for some participants, understandings shifted from absolute facts and clear right and wrong answers, towards negotiating and balancing different views. This required authentic opportunities for students to participate in dialogue.

One of the things I really valued about it was that it did bring together a whole bunch of people from all around the world from different disciplines all involved in different teaching activities… I found value in being able to kind of learn from them and ask relevant questions. So it wasn’t just about what I was getting from the presentations or lectures or necessarily the questions that came up during tutorials, it was more the learning community I was part of. (Participant 7-CE)

Enhanced understanding could generate a sense of agency, even as it increased the conceptual complexity of the field in which graduates were operating. Participant 6 commented that, before starting, applicants may not be in a position to understand what they are going to learn, nor the utility of learning about principles and theoretical frameworks to guide their own development of educational practices.

The types of transferable skills that I thought I would gain from the programme, were much more limited than the skills that I did gain… I think that I was under a common misconception, that I would learn a little bit more about some of the practical aspects of one on one education et cetera, and I think the focus was on the bigger picture and how things fit together, and the theoretical frameworks, and evidence that underpins some of the decisions that people make within medical education, and education in general, and I’m glad that it did. So, yeah, I think that the scope of my conception of education and teaching was much more limited and that my expectations fit within that more limited scope. (Participant 6-CE)

It may not be feasible to express the attributes that students gain from these programmes in ways that can be understood by those who have not yet been through the experience, since students may require an expanded, complex and nuanced view of education to grasp the possibilities and benefits of developing such a view.

### Field, habitus and capital

In the second phase of our analysis, we synthesised the themes above in relation to Bourdieu’s (1977, 1986) concepts of field, habitus and capital. We see our three themes as reflecting changes in habitus, because they convey durable changes in dispositions and capacities from which practices are adapted and developed and which, in turn, enhance the participants’ agency within their fields. Learning, then, involves developing the habitus that is required to successfully operate within a particular field. As Schatzki (2017, p. 29) noted, “the more the habitus is acquired, the better someone can proceed in these fields, and in a greater range of situations.” However, it is important to recognise that these capacities and dispositions are not simply acquired by individuals, they are caught up in other forms of capital that are brought about by understandings of the institution and accreditation of qualifications (*institutionalised capital*), as well as the social connections generated through enrolment and engagement with the programme (*social capital*).

The acquisition of a postgraduate degree in itself represents enhanced *cultural capital*: it is a valuable resource, as reflected in its common appearance in job descriptions and promotion criteria and its potential for mobilisation to progress a participant’s career and professional status.

Working here, I needed a postgraduate qualification… the fact that I am now theme head, [as a nurse] it’s quite unusual, so I think having the Masters has given me that credibility. (Participant 3-CE)

Graduates are inducted into their new status through rituals of graduation and the wearing of robes that denote the level of the qualification and the awarding institution. One is left in no doubt that one has graduated and entered into a community of highly-educated peers. The association with an internationally-recognised University (“Edinburgh is one of the top universities in the world”, Participant 16) also adds value in the form of *symbolic capital* (i.e. the attribution of particular qualities to that resource simply because of its source) (Bourdieu 1986). In the following excerpt, the symbolic capital that this particular institution lends its qualifications through its reputation and its grand estate is given priority over the accumulation of *embodied capital* (enhanced skills and knowledge).

It was a really nice moment to graduate from the University of Edinburgh, to be honest and candid; it’s a beautiful building we graduated in. I felt very proud of myself and even now, when I mention that’s where my master’s was from, you get a different look and you feel quite chuffed…. (P16-MP)

Participant 16 portrayed the intertwining of different forms of capital within the perceived value that the Master’s had for her, not just in terms of advancing in her field but also in economic terms:

You get respect, recognition, people listen to you… you know what you teach, you have confidence. You are not teaching just because you [have read] something… No, you lived it, you are the professional, you are qualified, certified… you are a resource. And definitely in terms of your income, you are more [highly] paid than others. This is obvious: for something where you have worked hard and sweat[ed], you have to be paid. (Participant 16-MP).

The value of a Master’s programme also related to the cost of studying, in terms of money, time, effort, and emotion. For Bourdieu (1986) embodied capital implies a personal investment of time, but the *appearance* of this investment also added to the available symbolic capital. Crucially, however, symbolic capital was, in turn, supported by the practical value of knowledge, attitude, and confidence. Thus, these Masters conferred “credibility” and “legitimacy” through a combination of skills and knowledge, status, social connections, and accredited qualifications.

What the master’s degree gave me really, was a bit of credibility, not only in name - people know I have it - but also in terms of skills and analysis and presenting arguments and scrutinising… (Participant 14-MP).

The different forms of capital could manifest not only as a greater sense of legitimacy, but also a greater actualisation of authority, a perceived competitive advantage, and an increase in effectiveness and influence. Indeed, developing an academic voice was sometimes seen as necessary for the development of effective practices and for career progression. Participant 5 described an occasion where her words were interpreted against the backdrop of the qualification.

I feel like it definitely made a difference in performing well at a Consultant interview.’ Cause they were all like, oh you’ve got an MSc, oh that’s great. Or you’ve got a distinction, well done… I think people recognise that coming out with a higher degree is… a firm commitment…. a really important bit of my CV that was impressive to people. Impressive enough to help me get the job that I wanted. (Participant 5-CE).

She felt that her “impressive” qualification, in combination with her knowledge, distinguished her in her field. Thus, cultural capital was enhanced not only by acquiring attributes, but also by having other people know about them. Another kind of social capital may have been even more important over the longer-term: valuable networks of supportive and influential colleagues were formed that lasted beyond the programme.

When we finally “met” in graduation, we felt that we belong to each other. We are truly classmates. We shared discussions and tried to remember our memories together which was wonderful. We felt that we are one family: we support each other, attend presentations for each other and we have… felt that we are not alone. We have family that support us.” (Participant 12-CE)

These networks could be mobilised in various ways that were advantageous to the individuals. However, becoming part of a network required the development of contextualised skills and practices, and a shift in understanding of the value of online learning communities. Early on in her studies, Participant 14, had reservations that her programme would not be “meaningful for [social connections], because you don’t meet people face to face.” Later, she came to recognise the value of social connections with people distributed across the world, indicating a need to develop more sophisticated conceptualisations of online interactions. The expanded worldview, generated through exposure to the variation in practices, disciplines and perspectives available within these online networks, created opportunities for thinking about new ways in which things could be done and, thus, the potential to change one’s habitus. Participant 2 commented on the benefit of comparing and contrasting practices with other students.

Meeting people who were doing essentially the same thing as me, but in much different settings and realising how it’s similar and how it’s different… it opened my eyes a little to how things could be done and are done in other places. (Participant 2-CE).

Such dialogue, developed and practised through these programmes, could have a lasting effect on practice. Further, by accruing symbolic, social and embodied capital through a Master’s qualification, and the knowledge and social connections that came with it, graduates could compensate—at least to some extent—for a lack of other kinds of cultural capital (wealth, prior education, social status, etc.). By completing the considerable challenge of postgraduate study, other graduates had transformed not only what they could do or how they were seen, but how they understood themselves and the nature of their professional role. This transformation can be seen as the development of habitus necessary to advance in academic and professional fields. For example, the combination of the forms of capital discussed so far enabled Participant 7 to overcome cultural obstacles and gain access to higher status and increased agency.

At the time that I chose general surgery, no girls or ladies were allowed to visit surgery in our university, it was a department for men only… I have exerted maybe…trebled the effort that my male fellows have exerted. That’s what made me get my Master’s degree, and I got the position of assistant lecturer in general surgery department at our university. … [This] got the professors and the senior staff to see that I can be a surgeon, it’s not for men only. I have opened the door for a lot of ladies to join the department afterwards. (Participant 7-CE)

Thus, significant agency could be required in order to amplify the capital derived from the qualifications. Another example was Participant 11, who actively lobbied for formal recognition of his qualification. He became “the first [doctor in his country] to get a Master’s degree in education,” opening up significant opportunities for career progression. It was interesting to see how the attributes gained through these Master’s could disrupt existing hierarchies, in minor and major ways. Participant 7 (above) demonstrated a significant shift in power and legitimacy, becoming recognised as a female surgeon in a male-dominated field. Participant 11 raised the profile of clinical education in his country. Participant 13 spoke of the Master’s qualification as necessary to accessing particular areas of the field of medical education that would otherwise be inaccessible to nurses.

## Discussion

While our interviews revealed evidence of all of the University of Edinburgh’s stated graduate attributes, and while those attributes are apparently positive (though potentially very demanding), a number of aspects remain unclear. Within the accounts of our participants, there was no obvious way in which either the programmes or the institution had actively developed these attributes. It is not clear why the University should claim and promote *these* particular attributes, rather than others that might also be claimed. This, we suggest, is an area for further research: how do programmes and institutions work towards their particular graduate attributes? How could we assess the extent to which graduates attributes are realised? Are they aspirational, in the sense that our graduates will continue to work towards them across their careers (and then is simply imbuing graduates with these aspirations sufficient, or should institutions and teachers be responsible for facilitating their development, and to what extent)?

Participants indicated various ways in which undertaking these online Master’s programmes had enhanced cultural and social capital, and changed habitus. Through developing an academic voice, infectious curiosity, and an expanded worldview, they had developed a range of resources that they could employ across their educational and clinical fields. Thus, our results suggest the development of attributes, through postgraduate study, that can be mobilised in professional contexts. However, these differ in important ways from conventionally-defined graduate attributes, including those explicitly claimed by the University of Edinburgh. For example, the development of infectious curiosity reflected the proposed graduate attribute that “*University of Edinburgh graduates have:* curiosity for learning that makes a positive difference.” However, it is useful to separate curiosity from positivity, since putting this constraint (albeit a positive one) on curiosity is counterintuitive. Curiosity does not predetermine whether enquiry will lead to a positive difference or, indeed, any difference at all. Considering the second attribute of “courage to expand and fulfil their potential,” some of our participants demonstrated courage (e.g. to argue for recognition or an increase in authority or status), and there was clear evidence of particular forms of “expansion” (e.g. of world view, responsibility, influence, social networks). However, it cannot be proven or refuted that participants had fulfilled their potential. Further, courage is only necessary when one does not have full confidence, and we found confidence—supported by the accumulation of various kinds of capital that allowed graduates to gain an advantage within their fields—to be a stronger theme in the data. It should, of course, be recognised that developing confidence is contingent on various forms of historic, cultural capital that applicants bring with them when starting a programme. It might be, then, that *courage* is required to surmount obstacles that come about through a shortfall in capital. Both *courage* and *passion* (“to engage locally and globally”) were required for some participants to successfully argue for the funding they needed to start their programmes, and for others to argue for their qualification to be officially recognised.

Indeed, cultural capital is more difficult to acquire for some than for others. As Bourdieu (1986) argued, those who arrive with significant cultural capital (e.g. in the form of family support, a privileged background, or a solid grounding in the language of the new context) are in a significantly advantageous position to acquire yet more cultural capital. Thus, while these Master’s programmes did help those who faced cultural obstacles (such as “being a nurse” or “being a woman”) to overcome them, it cannot be said to have levelled the playing field, since those who bring cultural capital with them may have an advantage in completing the programme and in using it afterwards to progress their career. Therefore, we believe that confidence is a more relevant attribute for these programmes, while courage is required for those facing obstacles to participation due to a shortfall in various forms of capital.

This discussion shows that it is simply too easy to argue that the stated attributes have been developed, because they are ongoing achievements without any clear metric or benchmark. In contrast, Bourdieu’s concepts can explain the actual or potential impact on practice in the graduate’s own field(s). Our results suggest that participants were transformed by their studies; often in ways they had not anticipated. Indeed, we argue that the most important outcomes of these programmes were only understood when looking back over the journey taken, and it may not be feasible to use the themes reported here as promotional devices, since applicants may appreciate neither the possibility nor the need for this development. Nonetheless, participants had developed capacities, and ways of thinking and practising (McCune and Hounsell 2005), that could help them progress in their work and in their careers.

Bourdieu (1986) saw educational qualifications as an *institutionalised* form of cultural capital which made them more easily convertible into economic capital. As legally-sanctioned and guaranteed certificates of “cultural competence” (p. 20), they are relatively independent of the knowledge and abilities of the graduate. They distinguish graduates from those who have learned through less formally-sanctioned means, but do not, by themselves, mean that the knowledge of graduates is superior to that of non-graduates. Nonetheless, institutionalised capital can allow graduates to act and be perceived *as if* their knowledge is superior, which in turn can advance their development in other areas.

Beyond the formal certificate, there are other ways in which postgraduate study can confer advantages. There is the actual learning of knowledge and skills in the form of *embodied cultural capital*. There are also the social connections, built up through the programme, that can continue beyond graduation. It is interesting to consider that, as their fellow graduates gain authority and status, our participants’ own social capital increases, since, according to Bourdieu (1986, p. 21), it “depends on the size of the network of connections he can effectively mobilize and on the volume of the capital (economic, cultural or symbolic) possessed in his own right by each of those to whom he is connected.” This suggests that it is in each student’s interest to be collaborative, rather than competitive, since the learning and enhancement of one’s peers is of potential value to oneself.

Bourdieu (1977) conceived of people (in our case, clinical educators) as vying against each other within a *field* for different forms of capital and status. However, we have shown that students can acquire capital within their field by using postgraduate programmes to learn about education, to obtain a relevant qualification, but also to generate valuable social networks. It is in this last issue that the greatest tension arises in Bourdieu’s position: significant value comes from the development of supportive partnerships and networks, and participation in a community of learners. Thus, our position, upon reflection on the findings presented here, is that graduates can become more competitive in their fields of clinical education and, indeed, clinical practice, by being more cooperative and collaborative within the site of their studies. It follows that postgraduate programmes should function not as sites of conflict, but of nurture and mutual support, and that may be best facilitated by pedagogies that actively work towards “lasting, useful relationships” and the fostering of a collaborative culture. We argue that postgraduate programmes will benefit from designs that actively encourage students to share experiences of professional practice and work with peers on problems that involve applying new knowledge to professional domains. Further, tutors and the institution should work actively to reduce perceived negative forms of hierarchy and competition between students (although we recognise the challenge that comes from assessment and the allocation of grades).

While Bourdieu’s concepts have been helpful in advancing our thinking, we do not wish to come across as overly cynical about the value or values of postgraduate education. While we have explored the acquisition of capital as a means to develop and exert individual agency within a field, we do not see the activities of our participants as entirely self-motivated, nor do we believe that it is always appropriate to emphasise self-motivation within what is a complex interplay of personal and social motivations. For example, when Participant 4 said that she was “very proud” of having used her Masters qualification to be appointed to be an examiner for “the next wave of new GPs,” we see this as *both* a self-motivated advancement of career, influence and status, *and* a socially-motivated aspiration to contribute to the advancement of the profession and those who are just beginning their careers. The political or social moves made by graduates to increase their power or capital in the workplace are not necessarily explicitly strategic or rational; they may be natural choices of a habitus that has preconditioned them in certain ways, of which they may be unaware (Bourdieu 1986). As discussed above, it is possible that the appropriation, through postgraduate study, of language and tools of reflection and analysis might make graduates more aware of these processes, and may empower them to manipulate them. It is for this reason that discrepancies between our results and the stated graduate attributes may come about: it is impossible to precisely define such outcomes realistically, because the complexity of definition exceeds the capacity of applicants to understand it (a problem that is only solved by studying).

Rather than attempting to determine how PGT programmes produce graduate attributes, it has been more meaningful to consider how changes in habitus and capital, emergent through and after PGT programmes, relate to desirable and durable changes in practice. This can form the basis for a more realistic and precise conceptualisation of graduate attributes. For example, we argue that “attributes” may be best seen as durable but not inherent or fully achievable. “Graduate,” then, could be taken as an indicator the person has gone through a formalised, structured process to begin the sustained (i.e. over the course of their career) development of that attribute. Our results support previous evidence of the transformative potential of postgraduate qualifications (Sethi et al. 2016, 2018). However, our theoretical perspective has also highlighted that Sethi and colleagues did not adequately consider the ways in which self-efficacy itself is contextual, or what is involved in maintaining self-efficacy across contexts. By considering how our participants’ developing agency manifested across clinical and academic settings, we advance understanding of the impact of online postgraduate programmes beyond identity and practice, offering an alternative explanation of the potential, interrelated and largely unpredictable benefits of PGT programmes.

### Limitations

Mindful of criticisms of single institution research, we acknowledge that we could learn more by extending the study to a wider range of institutions and disciplines. For example, we speculate that changes in habitus, capital and practice are likely to be different between healthcare and other fields, professional and non-professional education, and between undergraduate and postgraduate students. Thus, further studies in different contexts will help researchers gain a broader understanding of the transformative potential of different kinds of education under different conditions. However, we are also aware of the criticism that medical education research is theory-light (Albert et al. 2007) and this paper represents an application of Bourdieu’s theoretical framework to a small group of Master’s graduates from two online PGT programmes in order to shed some light on how the attributes that they develop through their studies manifest in their academic and clinical practice.

Another important limitation is that we did not directly examine the habitus or cultural capital that our participants arrived with at the start of their postgraduate studies, instead focusing on perceived changes in practice and agency brought about through their studies. Bourdieu (1984) recognised that education was one of the paramount ways that privilege can be transmitted, and thus education is imbued with forms of capital in which it makes sense to invest (Frank 2013). However, while our results suggest that such degrees are beneficial to graduates in economic, social and cultural terms, Master’s degrees bring considerable financial cost and are, therefore, outside of the reach of many. Indeed, there is some suggestion in the sociological literature that postgraduate qualifications are the new frontier of social mobility (Wakeling and Laurison 2017), and studies have shown that forms of capital valued within medicine, for example, are not equally accessible to all medical students, particularly those from lower socio-economic backgrounds (Mather and Parry 2009). Bourdieu has been criticised for assuming that cultural capital is experienced in similar ways (Sullivan 2001), and future research, incorporating an examination of how the social, cultural and economic backgrounds of students create challenges or opportunities for accessing and engaging in postgraduate education, could usefully extend his framework.

## Conclusion

In this paper, we have considered the experiences and perceptions of graduates of two online Master’s programmes in relation to changes in agency and practice across academic and professional healthcare settings. In many cases, despite their varied reasons for undertaking postgraduate study, our participants felt that the benefits obtained were greater, and of a different kind, from those they had anticipated when starting their programmes. Despite the small sample (17), our results provide potentially interesting insights into the value and utility of these qualifications for those who hold them. The perceived increases in confidence and agency of our participants were largely built on a combination of developed knowledge and, at the same time, an enhanced capacity to appreciate and deal with complexity and uncertainty. This capacity underpinned the pursuit of intellectual curiosity and the crossing of disciplinary and epistemological boundaries.

The online delivery of both programmes afforded powerful opportunities for international communication and interaction, as practitioners from around the world engaged in dialogue around the similarities and differences between settings and practices. This was highly valued by participants, indicating the potential to develop social capital through global networks, as well as possibilities for opening up each graduate’s practices for reflection and refinement. The three resultant themes of academic voice, infectious curiosity and expanded worldview all benefited from a collaborative and discursive approach to online education. Our work, therefore, has implications both for those designing and delivering these programmes, and for prospective applicants: programme design should encourage collaboration and discussion, and this aspect of effective postgraduate study should be made explicit to potential students.

Through Bourdieu’s concepts of field, habitus and capital, we have been able to demonstrate how the completion of a postgraduate qualification can be used to advance the individual in their own field (i.e. clinical or academic context) and to consider how graduate attributes manifest in practice. In doing so, we have proposed that graduate attributes, as typically conceived by universities, are not sufficiently contextualised. Not only is their manifestation context-dependent, but changes in habitus and capital are difficult, if not impossible, to pre-determine. Nonetheless, it is clear that such programmes can enhance graduates’ agency in complex and multifaceted ways across academic and clinical settings.
